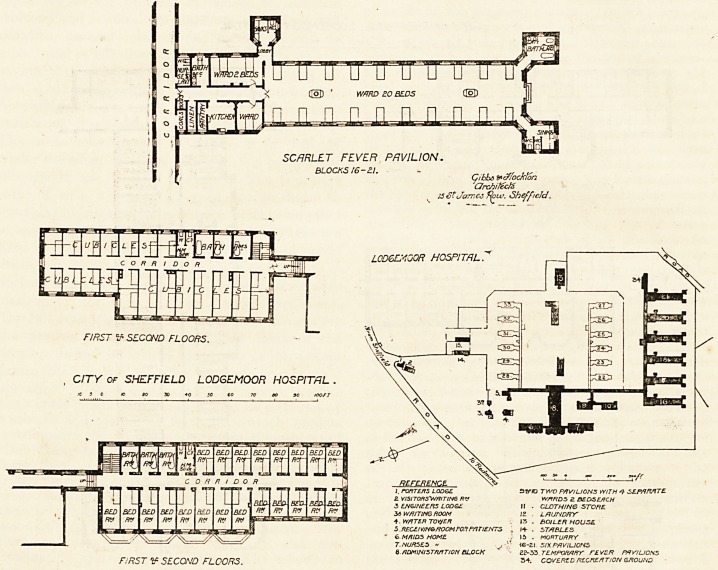# Hospital for Infectious Diseases, Sheffield

**Published:** 1904-02-06

**Authors:** 


### HOSPITAL ADMINISTRATION.

### CONSTRUCTION AND ECONOMICS.

### HOSPITAL FOR INFECTIOUS DISEASES, SHEFFIELD.

THIS hospital occupies a fine situation on Lodge Moor, about $4\frac{1}{4}$ miles from the centre of the town. The entire site extends to 40 acres and it is 900 feet above the sea-level. About 24 acres are given up to the various blocks constituting the hospital, and the rest of the land is used for agricultural purposes. The City Council are certainly deserving of praise for securing such an extensive and desirable site.

The hospital really originated in the year 1887, during the small-pox epidemic. The buildings were of a temporary nature and consisted of twelve pavilions accommodating a total of 150 or 200 patients. These pavilions are now used for scarlet-fever cases, and the new part, which may be called the hospital proper, consists of six fine permanent pavilions as well as isolation blocks and an administration department. The latter has on one side of the entrance hall the matron's room and the medical officer's office, and on the other side are the assistant medical officer's rooms. Behind these runs a corridor giving access on the right-hand side to the nurses' home, and on the left side to the maid's residence. Another corridor at right angles to this divides the administrative department into two, and ends in the main corridor, which in its turn communicates with three other corridors, two of which lead to the old temporary pavilions, and the other joins the new pavilions with each other, thus linking all the sections of the hospital into one, and yet leaving each block distinct from its neighbours.

The ground floor of the administrative department illustrates the enormous advantage of having plenty of land to build on, and it also shows the skill of the designers, for it contains every possible section and requirement. The kitchen is in shape rather more in length than twice its width, and it is admirably lighted by nine large windows. The nurses' dining-room and the maids' dining-room are both near the kitchen, so that the meals can be served with great ease. As regards the former, we are inclined to think it is almost too near, as we believe in the principle of keeping the two staffs of a hospital as distinct as possible, and proximity is rather a bar to this. We should prefer to have seen the nurses' dining-room in the nurses' block. The first floor of the administrative block to the front is approached by two staircases, one leading to the medical officers' bedrooms, and the other to the matron's rooms, and this part is very well arranged.

The nurses and the maids have their sleeping rooms chiefly on the first and second floors of their respective blocks, where also are provided several bath-rooms for them; and on the ground floors are their sitting-rooms and per bed. As every bed has a window on both sides this 2,000 feet ought to be sufficient even for infectious cases, always provided that the influx of fresh air is looked after, as much more depends on this than on mere cubic space. The bath-rooms, closets and sinks are quite correctly placed and arranged, and the blocks containing them are cut off from the wards by efficiently cross-ventilated passages. At the corridor end of the ward are the ward kitchen and other offices, as well as a single-bedded room and a double-bedded room. The former has a window in the angle as well as another window and so possesses a certain, or uncertain, amount of cross-ventilation by means of the window in the angle and by the door and fireplace. The latter has also a door and fireplace opposite

SCARLET FEVER PAVILION.

BLOCKS 16-21.

FIRST & SECOND FLOORS.

CITY OF SHEFFIELD LODGEMOOR HOSPITAL.

10 5 0 10 20 30 40 50 60 70 80 90 100 FT

FIRST & SECOND FLOORS.

their reading-rooms; as well as box-rooms, boot-rooms and lavatories.

The water tower stands near the administrative department. It is 84 feet high, and contains a tank holding 12,000 gallons of water. The water supply comes from the Corporation mains, and this tank is merely for reserve purposes or for use in case of fire. Near the water tower are the house for the engineer and a waiting-room. The north end of the main communicating corridor is close to, and projecting from it is the receiving-room for patients.

The six new blocks are alike. Each is 120 feet long and 26 feet wide, and is intended for 20 beds, giving, therefore, about 150 superficial feet per bed, and, assuming a ceiling height of 12 feet, the cubic space would exceed 2,000 feet the windows, but such cross-ventilation as it can obtain from these must be only into the corridor leading to the ward. Where much is so good in the planning we regret to have to notice these drawbacks, and it is strange how often this point of cross-ventilation of small wards is overlooked by architects.

The pavilions run north and south, which is by far the best aspect; indeed, the only proper one for hospital wards.

There is a covered playground provided for the children, and the mortuary occupies a good position between the north blocks and the gate-porter's lodge.

The entire accommodation is now for about 400 patients. The cost of the new buildings was £83,814 for 266 beds, being about £315 per bed. The architects are Messrs. Gibbs and Flockton, of Sheffield; and the contractor is Mr. T. Roper.

## Figures and Tables

**Figure f1:**
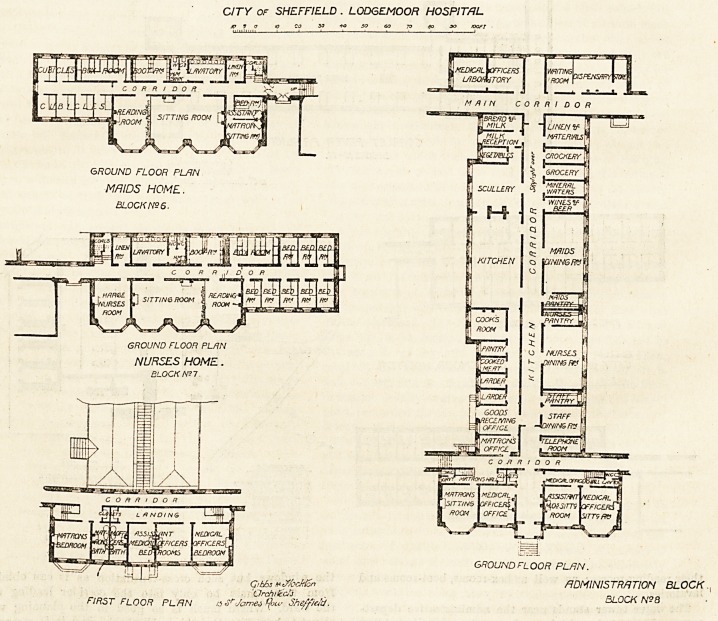


**Figure f2:**